# Zika en la gestación. Afectación de las destrezas de ejecución y edad madurativa en infantes*

**DOI:** 10.15649/cuidarte.1928

**Published:** 2022-10-12

**Authors:** Viviana Karina Hernández-Vergel, Raúl Prada-Núñez, César Augusto Hernández-Suárez

**Affiliations:** 1 Universidad de Santander, Facultad de Ciencias Médicas de la Salud, Grupo de investigación en biomecánica, Comunidad y neurodesarrollo - Entropía, Cúcuta, Colombia. E-mail: vivi.hernandez@mail.udes.edu.co Universidad de Santander Universidad de Santander Facultad de Ciencias Médicas de la Salud Cúcuta Colombia vivi.hernandez@mail.udes.edu.co; 2 Universidad Francisco de Paula Santander, Cúcuta, Colombia. E-mail: raulprada@ufps.edu.co Universidad Francisco de Paula Santander Universidad Francisco de Paula Santander Cúcuta Colombia raulprada@ufps.edu.co; 3 Universidad Francisco de Paula Santander, Cúcuta, Colombia. E-mail: cesarauguto@ufps.edu.co Universidad Francisco de Paula Santander Universidad Francisco de Paula Santander Cúcuta Colombia cesarauguto@ufps.edu.co

**Keywords:** Virus Zika, Gestación, Destrezas, Desarrollo Infantil, Zika Virus, Gestation, Abilities, Child Development, Zika Virus, Gestado, Habilidades, Desenvolvimento Infantil

## Abstract

**Introducción:**

El virus Zika se transmite por la picadura de mosquitos infectados, pero también puede ocurrir a través de una infección intrauterina antes del parto, y el virus pasa al feto. Objetivo: describir el nivel de afectación en destrezas de ejecución y edad madurativa de niños del programa Valientes del Futuro con la infección neonatal por virus Zika.

**Materiales y Métodos:**

La investigación se enmarca con enfoque cuantitativo de tipo correlacional apoyada con una investigación de campo y diseño no experimental, con una muestra de 15 infantes de 3, 4 y 5 años. La técnica de recolección utilizada fue la Escala Abreviada.

**Resultados:**

En cuanto a las áreas evaluadas con respecto a la edad madurativa se encontró que la ponderación de la destreza motora y praxis halló un coeficiente de correlación de 0,601 (moderada) y en la muestra de las destrezas de ejecución de los niños de 3 años con zika gestacional se obtuvo una correlación de 0,853 (fuerte).

**Discusión:**

la infección por virus zika en niños y niñas, adquirida durante la gestación, limita fuertemente las destrezas de ejecución propias de la edad madurativa en esta población.

**Conclusiones:**

Existe correlación entre los infantes con zika gestacional y la afectación fuerte en las acciones o comportamientos que un paciente tenga para moverse e interactuar físicamente con actividades, objetos y por ende realizar una actividad motora aprendida.

## Abstract (en)

**Introduction:**

Zika virus is transmitted through the bite of infected mosquitoes, but it can also occur through an intrauterine infection before delivery, and the virus passes to the fetus.

**Objective:**

describe the level of impairment in performance skills and maturation age of brave boys and girls of the future program with neonatal Zika virus infection.

**Materials and Methods:**

Research is framed with a quantitative correlational approach supported by field research and non-experimental design, with a sample of 15 infants aged 3, 4 and 5 years. The data collection technique used was the Abbreviated Scale.

**Results:**

Regarding the areas evaluated with respect to maturational age, it was found that the weighting of motor skills and praxis found a correlation coefficient of 0.601 (moderate) and in the sample of performance skills of 3-year- old children. With gestational zika, a correlation of 0.853 (strong) was obtained.

**Discussion:**

Zika virus infection in boys and girls, acquired during pregnancy, strongly limits the execution skills typical of maturational age in this population.

**Conclusions:**

there is a correlation between infants with gestational zika and strong involvement in the actions or behaviors that a patient has in moving and physically interacting with activities, objects and thus performing a learned motor activity.

## Resumo

**Introdujo:**

O virus Zika é transmitido pela picada de mosquitos infectados, mas também pode ocorrer através de infecgáo intra-uterina antes do nascimento, e o virus é passado para o feto.

**Objetivo:**

descrever o nivel de deficiencia nas capacidades de desempenho e maturidade das crianzas do programa Braves of the Future com infecgáo neonatal pelo virus Zika.

**Materiais e Métodos:**

A investigado é enquadrada com uma abordagem quantitativa de tipo correlacional apoiada por uma investigado de campo e desenho náo experimental, com uma amostra de 15 bebés de 3, 4 e 5 anos de idade. A técnica de recolha utilizada foi a Escala Abreviada.

**Resultados:**

Em termos das áreas avaliadas com respeito a maturidade, verificou-se que a ponderado da pericia motora e praxis encontrou um coeficiente de correlagáo de 0,601 (moderado) e na amostra de pericia de execugáo de crianzas de 3 anos com zika gestacional foi obtida uma correlagáo de 0,853 (forte).

**Discussao:**

A infecgáo com o virus zika em crianzas, adquirida durante a gravidez, limita severamente as capacidades de execugáo desta populado na maturidade.

**Condusóes:**

Existe uma correlagáo entre os bebés com a zika gestacional e a forte afectado nas acgóes ou comportamentos que um paciente tem de mover-se e interagir fisicamente com actividades, objectos e, portanto, realizar uma actividade motora aprendida.

## Introducción

El virus Zika es un Flavivirus que pertenece a la Familia Flaviviridae y fue descubierto en monos en el año 1947 en Uganda^1^^,^^2^ y posteriormente en humanos en 1952 en Uganda y la República Unida de Tanzania. Asimismo, los flaviviridae son virus de ARN de cadena positiva que incluyen patógenos humanos como el virus del Nilo Occidental, el virus de la fiebre amarilla, el virus del dengue transmitido por mosquitos, el virus de la encefalitis japonesa y el virus de la encefalitis transmitida por garrapatas.

Este, virus transmitido por artrópodos (arbovirus), tiene su vector en el mosquito Aedes aegypti; Es decir, se transmite por la picadura de mosquitos infectados, pero también puede ocurrir a través de una infección intrauterina. La transmisión congénita o intrauterina del virus del Zika ocurre cuando una mujer se infecta con el virus del Zika durante su embarazo, pero antes del parto, y el virus pasa al feto.

Al respecto, Foy y colaboradores indican que “la evidencia circunstancial sugiere la transmisión directa del virus de persona a persona, posiblemente sexual”^3^, aunque no descartan otras posi bilidades como el intercambio de otros fluidos corporales, incluida la saliva. Pero hasta la fecha no se encontrada evidencia confiable de transmisión del Zika al besarse. De igual manera, es necesario destacar que la infección por virus Zika (ZIKV) es asintomática en cerca de un 80% de los casos^4^^,^^5^. Por otra parte, aunque el virus Zika (ZIKV) se aisló desde hace décadas:

*comenzó a cambiar en 2013 cuando el aterrizaje del ZIKV en la Polinesia Francesa se asoció con mayores tasas de síndrome de Guillain-Barré, una condición autoinmune que afecta el sistema nervioso periférico y puede desencadenarse por infecciones. En 2015, solo dos años después del brote polinesio, se registró una epidemia sin precedentes en Brasil. Allí, el ZIKV se convirtió en una carga de salud extraordinaria debido a la nueva correlación entre este y las malformaciones ce rebrales graves en los recién nacidos.* (pag1)^6^

Queda claro un cambio importante en el comportamiento de la infección, evidenciado a partir de una mayor severidad en la presentación clínica y complicaciones asociadas como altera ción en el desarrollo cerebral fetal (provocando microcefalia) y síndrome Guillain-Barré. Por otro lado, las epidemias del zika fuera del continente africano (aparición del linaje asiático), se da después del año 2007 y es especialmente en el año 2015 cuando entra a América^7^^,^^8^.

En este sentido, Gomes y colaboradores revelaron que “a su entrada en América éste nuevo patógeno generó, sólo en Brasil, el reporte de 440.000 a 1.300.000 casos sospechosos y más de 4.000 casos de microcefalia posiblemente asociados, entre septiembre de 2015 y febrero de 2016”^9^. Según Galán y colaboradores existen “seis países, territorios y áreas que informan casos de microcefalia potencialmente asociados con la infección por el virus del Zika. Estos son Brasil, Cabo Verde, Colombia, Polinesia Francesa, Martinica y Panamá”^10^. Por ello, la adquisición de in fección durante el embarazo se ha convertido en un riesgo importante de malformación fetal, por lo que debe priorizarse en estrategias de control de su transmisión.

La Organización Panamericana de la Salud/Organización Mundial de la Salud^11^, muestran que, en Colombia cinco departamentos concentraron el 58% de los casos sospechosos y 53% de los confirmados en el lapso de las semanas 32 de 2015 a 52 del 2016: Norte de Santander, Valle del Cauca, Santander, Tolima y Huila, posicionando al Norte de Santander en el primer lugar a nivel nacional^12^.Con el agravante de una tasa de incidencia tres veces superior a la nacional (277,29 casos/100.000 habitantes), en el periodo comprendido entre las semanas 1-21 de 2016, de 765,72 casos/100.000 habitantes. El Departamento Norte de Santander continuó registrando casos, con 1 de los 3 casos reportados desde el 01 de enero al 03 de junio de 2017, (junto con Tolima y Risaralda)^13^.

De igual modo, en lo referente a gestantes (población susceptible de complicación), se registraron 16.323 casos, de los cuales 5.420 se confirmaron por laboratorio (33,2%), como consecuencia de la epidemia en Colombia. Dentro de este último grupo, 1.203 (19%) provenían de Norte de Santander, con una mortalidad fetal y perinatal asociada del 5,96% del total de embarazos culminados, con un predominio de aborto (68,3%) debido a infección durante el primer trimestre.

Por ende, esta indagación ofrece un avance científico en torno al virus del Zika, puesto que, este virus pudiera comprometer al sistema nervioso, tanto a nivel central (cerebro) como a nivel periférico (nervios).En estudios realizados, mencionan que un porcentaje bajo de personas infectadas desarrolla las complicaciones neurológicas como se mencionó en apartados anteriormente. Por lo tanto, el zika relacionado a los casos de mujeres embarazadas, uno (1) al 13% puede presentar alteraciones neurológicas en el feto, por ello, esta investigación añade un diferencial con otras exploraciones, las cuales tuvieron como principal desafío la confirmación diagnóstica y estudio de los mecanismos inmunológicos que conducen al Guillain-Barré.

En lo práctico, investigar sobre el virus zika durante gestación y la afectación de las destrezas de ejecución y la edad madurativa de niños y niñas de madre gestante cuyo embarazo se desarrolló entre 2015 y 2016 durante la fase epidémica del Virus Zika, atendida en el Hospital Universitario Erasmo Meoz de la ciudad de Cúcuta y considerada como caso de Zika gestacional.

Las situaciones problemáticas indicadas anteriormente, así como su justificación respectiva, permiten formular las siguientes interrogantes de investigación: ¿Se identifica, los antecedentes de infección por virus Zika tanto individuales como maternos durante gestación mediante la revisión de historias clínicas en el programa Valientes del Futuro? ¿Se determina, la edad madurativa y las destrezas de ejecución de los niños y niñas priorizados del Programa Valientes del Futuro y población control mediante Escala Abreviada de Desarrollo y lista de chequeo? Estas interrogantes menores podrán dar respuesta a una mayor: ¿se puede describir, el nivel de afectación en destrezas de ejecución y edad madurativa de niños y niñas del programa valientes del futuro con la infección neonatal por virus Zika?

### 1.1. El virus zika durante gestación. Afectación de las destrezas de ejecución y la edad madurativa de niños y niñas.

#### 1.1.1. Gestación y Zika

Para Bolaños^((^^14^^))^ el embarazo o gestación:

...es el estado fisiológico de la mujer por el que, a lo largo de 281 días como media, se de sarrolla en su seno un nuevo ser humano. El embarazo se considera una etapa anabólica, en la que la creación de tejidos nuevos conlleva un aumento ponderal progresivo (10 kg de media). Este aumento de peso está condicionado por: feto (3.400 gramos [g]), placenta (650 g), líquido amniótico (800 g), líquido extracelular (1.680 g), otros tejidos y reserva de grasa para asegurar la lactancia (3.345 g), útero y mamas (1.375 g) y sangre (1.250 g) (pag1197)

Asimismo, Lugones y Ramírez^15^, definen el embarazo:

*al período que transcurre entre la implantación en el útero del óvulo fecundado y el momento del parto. Comprende todos los procesos fisiológicos de crecimiento y desarrollo del feto en el interior del útero materno, así como los significativos cambios fisiológicos, metabólicos e incluso morfológicos, que se producen en la mujer encaminados a proteger, nutrir y permitir el desarrollo del feto.*

Como resultado, casi todos los miembros y sistemas de la gestante experimenta alteraciones anatómicas y físicas que se revierten en el puerperio. Aunque, ciertos neonatos nacen con complicaciones de infección en el embarazo. Belfort^16^ habla que la infección por virus Zika se ha relacionado con la aparición de alteraciones neurológicas en recién nacidos debidas al especial neurotropismo que presenta el virus. Por otro lado, que en lo concerniente al diagnóstico para Gourinat^17^, el ZIKV puede detectase en sangre total, plasma, suero, orina, líquidos cefalorraquídeo y amniótico, semen y saliva. Al parecer puede ser detectado a más largo plazo en semen y orina que incluso en sangre.

Por ello, la Sociedad Española de Ginecología y Sociedad Española de Infectología Pediátrica (SEGO-SEIP) advierten que “el riesgo de que la infección por el virus Zika en una gestante pueda producir trastornos en el desarrollo del sistema neurológico del feto”^18^. También, estos autores señalan que el diagnóstico de infección congénita por virus Zika, se establecen los criterios epidemiológicos, clínicos y de laboratorio. Donde, el criterio epidemiológico para diagnosticar si el feto recién nacido de madre con antecedente es por si ha sido por infección por virus Zika; así como haber viajado o residido en zona de transmisión del virus durante el embarazo o haber mantenido relaciones sexuales sin protección con hombres diagnosticados de infección por virus Zika.

Mientras que el criterio clínico, refieren si el feto o recién nacido presenta microcefalia; anomalías de la neuroimagen, alteraciones neurológicas, afectación ocular, crecimiento intrauterino retrasado u otros hallazgos: Pies zambos, artrogriposis secundaria a daño neurológico de origen central.

Entretanto, que los criterios de laboratorio, refieren a los casos con detección de ácido nucleico por PCR en muestra clínica (suero, orina, LCR o Líquido amniótico); IgM positiva confirmada con anticuerpos neutralizantes en suero positivos; Persistencia de IgG positiva para Zika más allá de los 18 meses de vida. La presencia de anticuerpos IgM, no confirmada por neutralización en una muestra de suero, y, persistencia de la IgG entre los 6 y los 18 meses de edad (al menos dos muestras con concentración similar de IgG).

De igual modo, la Sociedad Española de Ginecología y Sociedad Española de Infectología Pediátrica (SEGO-SEIP), menciona que:

*publicación científica en la que se analizó una cohorte de 1.850 embarazadas infectadas con virus Zika en Colombia, concluyó que más del 90% de las mujeres que se habían infectado durante el tercer trimestre del embarazo habían dado a luz a recién nacidos que no presentaban ninguna anormalidad, incluida microcefalia. Sin embargo, en Brasil, la aparición de un rash en la gestante durante el tercer trimestre se asoció con alteraciones cerebrales a pesar de que los recién nacidos tenían perímetros craneales normales (50); también se identificaron 4 casos de microcefalia en hijos de mujeres asintomáticas (51,52)*^18^

Con base a los estudios existe el consenso científico para afirmar que la infección por el virus Zika es una causa de microcefalia y otras alteraciones neurológicas en recién nacidos. así como, el síndrome de Guillain Barré^19^. Al respecto, Arroyo sostiene "...que esta infección arboviral se asocia con un aumento de la incidencia de microcefalia en los fetos y niños nacidos de madres infectadas. El riesgo de microcefalia fetal es mayor para las infecciones que ocurren en el primer trimestre”^20^.

Este último autor argumenta que los niños con microcefalia pueden tener diferentes problemas como discapacidad intelectual, retraso del desarrollo, epilepsia, parálisis cerebral, así como trastornos oftalmológicos y auditivos. Asimismo, indicó que en un estudio de 680 niños informó que 65% de los niños con microcefalia tenían discapacidad intelectual, 43% epilepsia, y 30% afecciones oftalmológicas. La microcefalia aislada e idiopática familiar no se ha asociado con disminución de los resultados del desarrollo o puntuaciones de CI en niños sin otros déficits notables.

Por otra parte, el Ministerio de Salud de Argentina señala que "diversas infecciones, producidas por arbovirus, que pudiera afectar las destrezas de ejecución y la edad madurativa de niños y niñas”^21^, y, por ende, la adquisición progresiva de habilidades es la tarea primordial del sistema nervioso y es al reflejo de esta maduración a lo que se denomina desarrollo.

#### 1.1.2. Desarrollo madurativo

El desarrollo del niño está ligado al crecimiento. El crecimiento inicia en el momento de la concepción del ser humano y continúa a través de la gestación, la infancia, la niñez y la adolescencia. Por ello, el crecimiento es inherente al desarrollo. Ahora bien, el desarrollo involucra la madurez y refiere a la adquisición de destrezas y habilidades en varias etapas de la vida. De este modo, la maduración enfatiza la importancia de la naturaleza o la genética en el desarrollo humano, en oposición a la crianza o el medio ambiente.

El desarrollo de la maduración se produce en secuencias o etapas fijas que se rigen por los genes. Este "plan genético” para el desarrollo determina la secuencia, el momento y la forma de los patrones de acción emergentes^22^. Para ello, es necesario conocer adecuadamente las características de normalidad. Iceta y Yoldi^23^, menciona que el desarrollo normal se debe a la adquisición de ciertas habilidades, entre ellas se encuentra: La motricidad gruesa, para que el acto motor voluntario evolucione con normalidad hace falta que se produzca, por una parte, una progresiva diferenciación de los actos amplios e indiferenciados a otros precisos y concretos, que haya una proyección céfalo caudal y que además suceda de axial a distal.

Por otro lado, Escudero señala que "el niño es un sujeto que sigue un proceso de desarrollo altamente complejo. Por una parte, hay que considerar el desarrollo evolutivo de adquisición de funciones adaptativas e instrumentales. Este desarrollo se organiza en cuatro áreas principales: psicomotricidad, inteligencia, lenguaje y socialización”^24^.

Al respecto, la Organización Panamericana de la Salud citado por el Ministerio de la Salud de Argentina^21^ que las etapas evolutivas de la maduración refieren a las áreas: Motora, coordinación, lenguaje y social. a saber:

- Área motora: El desarrollo motor son céfalo-caudal, próximo-distal y de actividades globales específicas, es decir, que está relacionada a los principios de movimientos, que en los primeros meses de nacimiento son bruscos, amplios e incoordinados, aunque más tarde estos movimientos aparecerán lentos, limitados y coordinados, lo cual da el control a la motricidad gruesa, que son los movimientos que comprenden grandes áreas del cuerpo (sentarse, caminar, correr) y luego se irán adquiriendo el control de la motricidad fina (platear pelota, dibujar, escribir, y desarrollar así la capacidad de autonomía e independencia). En la motricidad fina, el desarrollo de la mano se aprecia desde los 4 meses aproximadamente y termina con la consecución del trípode manual (postura que adoptan los dedos pulgar, índice y medio para sostener el lápiz) allá entre los 4 y 6 años. Es necesaria la desaparición de los reflejos tónico flexor de la mano y la reacción tónico cervical asimétrica y lograr una coordinación con la vista para que la manipulación comience. Así pues, inicialmente sujetará un objeto colocado en su mano, para después ser capaz de buscarlo y alcanzarlo.
- Área coordinación: Refiere a la integración de funciones sensorio-motrices antes objetos y situaciones, es decir, coordinación audiovisual y óculo manual. La primera, permite buscar con la mirada las fuentes de sonidos. La segunda, permite desarrollar procesos de la presión antes los objetos que se encuentran a su alcance. Luego más tarde, se adquieren destrezas manuales complejas (introducir objetos pequeños en frascos o botellas), abrir o cerrar puertas, pasar hojas de un libro, vestirse y desvestirse, entre otras, cuando la flexión de la muñeca y la rotación del antebrazo comienzan a desarrollarse.
- Área Social: Hace referencia a las reacciones del niño ante el medio en que vive, así como la relación con la madre y con otras personas. La capacidad de integración y adaptación al ambiente (jugar, alimentarse y vestirse), las conductas de interacción, el proceso de socialización, individualización, autonomía e independencia. Por ello, en el desarrollo social, el niño irá adquiriendo unos patrones de conducta que le servirán para su interacción con el medio, porque por naturaleza es un ser social.
- Área Lenguaje: La utilización del lenguaje supone un canal de comunicación exclusivo de la especie humana que se pone en marcha en el primer año de vida. El lactante tiene muchas formas de comunicación preverbal: riendo, gritando y por rabietas; extendiendo los brazos para que lo cojan, cerrando la boca al ofrecerle comida. Entonces, esta área, refiere a toda forma de comunicación audible o visible (las miradas, gestos, percepciones del sonido y expresiones verbales), sean movimientos posturales, vocalizaciones, palabras u oraciones. En el mismo se incluye la imitación, la comprensión y el lenguaje articulado.

Todo lo anteriormente expuesto, induce que el desarrollo madurativo del recién nacido con infección por Zika dependerá de la afectación encontrada en las destrezas de ejecución de los infantes. Porque la maduración y crecimiento del individuo va de la mano con la parte cognitiva, social, afectiva, lenguaje adquiriendo estas habilidades según la edad que tengan ya que cada niño es un mundo diferente, donde la Escala Abreviada de Desarrollo podría ser una herramienta para identificar tempranamente anomalías motoras, de lenguaje, sociales y de coordinación.

#### 1.1.3. Escala Abreviada del Desarrollo - EAD

Para Alarcón y Trujillo^25^ el niño y la niña cuentan con capacidades físicas, psicológicas y sociales, como bases de sus procesos de interacción consigo mismo, con el mundo y con los otros. Siendo entonces el desarrollo durante la infancia un proceso complejo y de permanente cambio, que para cada niño y niña sucede de manera diferente, teniendo en cuenta las particularidades de sí mismo y su contexto. Por otra parte, la falencia en el desarrollo es un concepto que se emplea para evaluar a un niño o niña que no alcanza los hitos de desarrollo esperados para su edad son realizada desde la Escala Abreviada de Desarrollo - EAD^26^, incluso después de considerar la amplia variación de la normalidad.

Desde esta perspectiva, para Ortiz, la EAD, “es un instrumento diseñado para realizar una valoración global y general de determinadas áreas o procesos de desarrollo”^26^ que posibilita detectar alteraciones o problemas en los niños y niñas. Esta escala tiene como objetivo identificar de forma temprana, el riesgo de rezagos en el desarrollo de niños y niñas colombianos e hispanohablantes hasta los 5años.

El mismo se aplica desde el rango de edad, el ítem o pregunta sobre el área en cuestión: a) motricidad gruesa; b) motricidad fino-adaptativa, c) audición-lenguaje y; d) personal social. De este modo, se solicita que se anote edad en meses para cada evaluación. Los cuales dan cuenta de parámetros normativos para la evaluación del desarrollo de niños menores de 60 meses de alertas, para remitir al infante a una valoración médica.

Parafraseando a Ortiz^26^ sobre las áreas del desarrollo. El área de motricidad gruesa refiere a la maduración neurológica, control de tono y postura, coordinación motriz de cabeza, miembros, tronco. Mientras que el área motriz fino-adaptativa, hace referencia a la capacidad de coordinación de movimientos específicos, coordinación intersensorial: ojo-mano, control y precisión para la solución de problemas que involucran aprehensión fina, cálculo de distancias y seguimiento visual.

Entretanto, el área audición-lenguaje, implica la evolución y perfeccionamiento del habla y el lenguaje: orientación auditiva, intención comunicativa, vocalización y articulación de fonemas, formación de palabras, comprensión de vocabulario, uso de frases simples y complejas, nominación, comprensión de instrucciones, expresión espontánea. Y, el área personal-social, refiere a los procesos de iniciación y respuesta a la interacción social, dependencia-independencia, expresión de sentimientos y emociones, aprendizaje de pautas de comportamiento relacionadas con el autocuidado.

De este modo, utiliza materiales básicos para la administración de dicha escala y los cuales deben ser sencillo por la edad cognitiva de los infantes, tales como: Lápices o lapiceros rojo y negro; pelota de caucho de tamaño mediano, aproximadamente de 15 cms de diámetro; espejo mediano, caja pequeña de cubos de madera de aproximadamente 2cms. De lado (preferentemente 3 rojos, 3 azules y 4 amarillos); tijeras pequeñas de punta roma, juego de taza y plato de plástico, entre otros materiales.

Así como también, unos formularios para la observación y registro de la información sobre las áreas: De motricidad gruesa, motriz fino-adaptativa, de audición y lenguaje y, de personal social para evaluar las destrezas de ejecución, las cuales son las habilidades de desempeño que trabaja los terapistas ocupacionales.

#### 1.1.4. Destrezas de ejecución

Según la American Occupational Therapy Asociation^27^, se han utilizados múltiples ajustes que permiten catalogar y calificar las adaptaciones desde el abordaje de la terapia ocupacional frente a las exploraciones y prácticas acerca de la capacidad de habilidades en el desempeño eficaz de los infantes. Para Ares “las habilidades y destrezas son elementos que constituyen las actividades de la vida diaria en su aspecto familiar, social y laboral”^28^.

De acuerdo con lo citado anteriormente los profesionales de Terapia Ocupacional se enfatizan en comprobar e investigar cada una de las habilidades de ejecución con el propósito de saber todos aquellos aspectos que aparecen y apoyan o limitan el desempeño al instante de demostrar una de las actividades de la vida diaria. Al respecto, Anderson y Prada menciona que, en el marco para la Práctica de Terapia Ocupacional, se define a las habilidades o destrezas de ejecución como:

*“acciones observables, concretas y dirigidas hacia una meta que emplea la persona para participar en las actividades de la vida diaria. Las mismas pueden ser observadas mientras la persona desempeña tareas significativas en un entorno especifico, poniendo en juego y combinando las diferentes funciones y estructuras corporales. Las habilidades o destrezas de ejecución se clasifican en habilidades motoras, de procesamiento y de interacción y comunicación”*^*29*^

De este modo, las destrezas de ejecución que son actividades demostrables, donde sus categorías están interrelacionadas e incluyen:

*“Destrezas motoras y praxis: las motoras; son acciones que utiliza un cliente para moverse, incluye la planificación, secuenciación y ejecución de movimientos nuevos. Mientras que la praxis; son movimientos intencionales habilidosos, es la habilidad para realizar una actividad motora aprendida. Destrezas sensoriales-perceptuales: son las acciones o comportamientos que utiliza un cliente para localizar, identificar y responder a sensaciones y para seleccionar, interpretar, asociar, organizar y recordar eventos sensoriales basados en la discriminación de experiencias. Destrezas de regulación emocional: acciones que utiliza un cliente para identificar, manejar y expresar sus sentimientos mientras participa en actividades o interacciona con otros. Destrezas cognitivas: acciones que utiliza un cliente para planificar y gestionar el desempeño de una actividad. Destrezas de comunicación y sociales: acciones que utiliza un cliente para comunicarse e interaccionar con otros en un ambiente interactivo”*^30^

En consecuencia, se deduce que las destrezas de ejecución son acciones, movimientos que son asimiladas y acomodados a través del tiempo y situados en ambientes determinados, En el caso particular, de los niños de 3 a 5 años, que han sido infectados por ZIKA desde la gestación, las acciones o movimientos están relacionadas para llevar a cabo la ejecución de una destreza cuando estos la realicen, de acuerdo con el contexto y a las demandas de la actividad en el desempeño de la ocupación. Por todo lo anteriormente expuesto es que con este trabajo se pretendía describir el nivel de afectación en destrezas de ejecución y edad madurativa de niños del programa Valientes del Futuro con la infección neonatal por virus Zika, con el fin de evaluar dichas destrezas de acuerdo con prácticas de desempeño según edad cognitivas desde la terapia ocupacional.

## Materiales y Métodos

### El método

La presente investigación se enmarca en una investigación analítica con enfoque cuantitativo de tipo correlacional. El enfoque cuantitativo de acuerdo con Hernández, Fernández y Baptista “utiliza la recolección de datos para probar hipótesis con base en la medición numérica y el análisis estadístico, con el fin establecer pautas de comportamiento y probar teorías”^31^. Por otra parte, para Bernal, la investigación de tipo correlacional “tiene como propósito mostrar o examinarla relación entre variables o resultados de variables”^32^. Es decir, “conocer la relación o grado de asociación que exista entre dos o más conceptos, categorías o variables en una muestra o contexto en particular”^31^.

La investigación a su vez se inscribió dentro del tipo de estudio de campo. ya que “los datos de interés son recogidos en forma directa de la realidad”^33^ que en nuestro caso son los hijos nacidos de madres que durante su embarazo fueron o no afectadas por el zika.

De este modo, se espera realizar una completa caracterización de la población objeto de estudio para identificar la posible relación entre la afectación del zika de la madre en su proceso de embarazo y la presencia de algunas afectaciones en el desempeño en sus hijos del programa Valientes del Futuro de la ciudad de Cúcuta.

### Diseño de estudio

En la presente investigativa podría definirse como la investigación de diseño no experimental porque “se realizan sin la manipulación deliberada de variables y en los que sólo se observan los fenómenos en su ambiente natural para analizarlos”^31^. En la cual se buscó describir relaciones entre dos o más categorías, conceptos o variables en un momento determinado, que en nuestro caso son los hijos nacidos de madres que durante su embarazo fueron o no afectadas por el zika.

### Hipótesis operativas

Según Hernández, Fernández y Baptista^31^ las hipótesis correlacionales especifican las relaciones entre dos o más variables. Por ello, se plantea las siguientes suposiciones:

Hipótesis Nula (H_0_): La infección por virus zika en niños y niñas, adquirida durante la gestación, limita las destrezas de ejecución propias de la edad madurativa.
Hipótesis Alternativa (H_1_): La infección por virus zika en niños y niñas, adquirida durante la gestación, no limita las destrezas de ejecución propias de la edad madurativa.
Hipótesis de investigación de relación: Las variables, infección por virus zika en niños y niñas, adquirida durante la gestación y las destrezas de ejecución propias de la edad madurativa.

### La muestra

El tipo de muestra es no probabilística, dado que la elección de los elementos no depende de la probabilidad, sino de causas relacionada con las características de la investigación. En lo que respecta a la población objeto de estudio vale la pena aclarar que para poder realizar la valoración del infante es necesario contar con el consentimiento informado por parte de los padres, dada su condición de ser menores de edad y en condiciones especiales y se incluyeron con los siguientes criterios de selección:

*a) Inclusión (cohorte estudio)*

Niño o niña, hijo (a) de madre gestante cuyo embarazo se desarrolló entre 2015 y 2016 durante la fase epidémica del Virus Zika, atendida en el Hospital Universitario Erasmo Meoz de la ciudad de Cúcuta y considerada como caso de Zika gestacional.

Niño o niña de 3 a 5 años pertenecientes al programa Valientes del Futuro, con el antecedente materno mencionado.

Niño o niña considerado caso de Zika congénita, según historia clínica.

*b) Inclusión (cohorte control)*

Niño o niña, escolares de 3 a 5 años pertenecientes al Colegio Jaime Prieto Amaya sin antecedente inmunológico de infección por Virus Zika, ni antecedente de patología congénita o adquirida diagnosticada.

### Técnicas de Recolección de Datos

Según Arias, se entiende por técnica de investigación “el procedimiento o forma particular de obtener datos o información”^34^. En consecuencia, para el presente estudio se utilizó la Escala Abreviada del Desarrollo (EAD-1), prueba diseñada por el Ministerio de Salud de Colombia y el auspicio de Unicef.

El mismo, está compuesta por 120 reactivos de respuesta dicotómicos distribuidos en cuatro grupos de 30 ítems para evaluar la motricidad gruesa, motricidad fina, lenguaje y personal social^26^. La cual utiliza los siguientes parámetros normativos para la evaluación del desarrollo de niños menores de 108 meses (Ver Tabla 1)

La información validada se exportó al paquete estadístico SPSS versión 25 para el procesamiento de los datos. La base de datos fue almacenada en Mendeley Data^35^.

**Tabla 1:** Parámetros normativos para la evaluación del desarrollo de niños menores de 108 meses

Te a Edad en Meses	Motricidad Gruesa (A)				Motricidad Fina ( B)				Audición y Lenguaje (C)				Personal Social (D)				Total			
	Alerta	Medio	Medio Alto	Alto	Alerta	Medio	Medio Alto	Alt o	Alerta	Medio	Medio Alto	Alto	Alerta	Medio	Medio Alto	Alto	Alerta	Medio	Medio Alto	Alt o
1-3	0-1	2-3	4-5	6+	0-1	2-3	4-5	6+	0-1	2-3	4-5	6+	0-1	2-3	4-5	6 +	0-6	7-13	14-22	23
4-6	0-4	5-6	7-9	10+	0-4	5-6	7-9	10+	0-4	5-6	7-9	10+	0-4	5-6	7-9	10+	0-18	19-26	27-34	35
7-9	0-7	8-9	10-12	13+	0-7	8-9	10-12	13+	0-7	8-9	10-12	13+	0-7	8-9	10-12	13+	0-30	31-38	39-46	47
10-12	0-10	11-12	12-15	16+	0-10	11-12	12-15	16+	0-10	11-12	12-15	16+	0-10	11-12	12-15	16+	0-42	43-50	51-58	59
13-18	0-13	14-15	16-18	19+	0-13	14-15	16-18	19+	0-13	14-15	16-18	19+	0-13	14-15	16-18	19+	0-54	55-62	63-70	71
19-24	0-16	17-18	19-21	22+	0-16	17-18	19-21	22+	0-16	17-18	19-21	22+	0-16	17-18	19-21	22+	0-66	67-74	75-82	83
25-36	0-19	20-21	22-24	25+	0-19	20-21	22-24	25+	0-19	20-21	22-24	25+	0-19	20-21	22-24	25+	0-78	79-86	87-94	95
37-48	0-22	23-24	25-27	28+	0-22	23-24	25-27	28+	0-22	23-24	25-27	28+	0-22	23-24	25-27	28+	0-90	91-98	99-106	107
49-60	0-25	26-27	28-30	31+	0-25	26-27	28-30	31+	0-25	26-27	28-30	31+	0-25	26-27	28-30	31+	0-102	103-110	111-118	119
61-72	0-28	29-30	31-33	34+	0-28	29-30	31-33	34+	0-28	29-30	31-33	34+	0-28	29-30	31-33	34+	0-114	115-122	123-130	131
74-84	0-31	32-33	34-36	37+	0-31	32-33	34-36	37+	0-31	32-33	34-36	37+	0-31	32-33	34-36	37+	0-126	127-134	135-142	143
85-96	0-34	35-36	37-39	38+	0-34	35-36	37-39	38+	0-34	35-36	37-39	38+	0-34	35-36	37-39	38+	0-138	139-146	147-154	155
96-108	0-37	38-39	40+		0-37	38-39	40+		0-37	38-39	40+		0-37	38-39	40+	0-150	152-158			

De igual manera, se utilizó ficha de observación para la evaluación las destrezas de ejecución en las áreas de destrezas motoras y praxis, destrezas sensoriales perceptuales, destrezas cognitivas, destrezas de regulación emocional, destrezas de comunicación y sociales. La cual consta de 23 ítems para los niños de 3, así como también, la ficha de observación de 4 años refleja 36 ítems y, por último, la ficha de observación de 5 años está compuesta por 37 ítems, todas de respuestas dicotómicas.

De este modo, al confirmar el antecedente del virus Zika tanto individual como materno (en la corte de estudio), según la historia clínica (como se especificó), y la ausencia de antecedentes de infección por este agente en la cohorte control, se procedió a la valoración de las destrezas de ejecución mediante la lista de chequeo por edad de 3, 4 y 5 y la Escala Abreviada del Desarrollo. Para la cual se determinó la edad madurativa de cada participante en base a los hallazgos de la actividad anterior. Para finalizar se realizó el Diligenciamiento de base de datos con información obtenida para su vez hacer el análisis estadístico.

## Resultados

En los instrumentos aplicados se obtuvieron los siguientes hallazgos relacionados con los antecedentes de infección por virus zika tanto individual como materno durante la gestación, así como la edad madurativa y destrezas de ejecución, en el cual se conoció que el 60,0% de los infantes pertenece al sexo femenino y el 40,0% al sexo masculino, de los cuales 66,7% (10 infantes) tienen una edad cronológica de 3 años, un 13,3%(2 niños) se encuentra entre los 4 años y el resto un 20,0% (3 niños) tienen 5 años. Asimismo, se demostró que el 40,0% de las madres de los infantes pertenece a grupo especial de atención (desplazados).

Con respecto, a la infección por virus zika durante la gestación, se logró establecer que los infantes nacidos se relacionan especialmente con el antecedente, que la totalidad de dichos infantes tuvieron secuela de infección por zika gestacional y arrojaron un diagnóstico en trece de ellos (86,7%) de microcefalia, junto a otros diagnósticos tales como retraso de desarrollo (40,0%), Síndrome de Steven Johnson (13,3%), epilepsia (33,3%) y parálisis cerebral (33,3%).

Siendo este, el punto de partida para relacionar si el virus Zika al generar defectos congénitos asociados pudieran tener una afectación en el desempeño del cliente y con ello, las habilidades, características o creencias que residen en el cliente de acuerdo a su edad madurativa (Ver Tabla 2).

**Tabla 2:** Edad madurativa de los infantes con Zika Gestacional.

Edad cronológica	Meses	Rango de edad en meses (EAD)	Ubicación en los ítems de la (EAD)	Parte A- motricidad gruesa	Parte B- motricidad fina	Parte C- audición y lenguaje	Parte D- personal social	Total
3 años y 6m	42	37 - 48	22-23-24	11	11	13	17	52
3 años	36	25 - 36	19-20-21	7	4	7	5	23
3 años y 9m	45	37 - 48	22-23-24	6	4	7	6	23
3 años	36	25 - 36	19-20-21	9	0	0	0	9
3 años y 2m	38	37 - 48	22-23-24	6	4	7	7	24
5 años 4m	64	61 - 72	28-29-30	8	9	9	9	35
3 años y 7m	43	37 - 48	22-23-24	9	4	7	10	30
3 años y 6m	42	37 - 48	22-23-24	7	9	7	10	33
3 años y 8m	44	37 - 48	22-23-24	9	6	9	10	34
3 años	36	25 - 36	19-20-21	5	6	9	9	29
3 años y 6m	66	61 - 72	28-29-30	4	4	10	4	22
5 años	60	49 - 60	25-26-27	9	6	9	9	33
4 años y 2m	50	49 - 60	25-26-27	11	11	13	17	52
4 años	48	37 - 48	22-23-24	10	11	13	17	51
5 años y 2m	62	61 - 72	28-29-30	9	6	9	9	33
			Ítem mín.	4 meses	0 mes	0 mes	0 mes	
			de meses Ítem máx. de meses	11 meses	11 meses	13 meses	17 meses	
			Moda	9 meses	4 meses	7 meses	9 meses	

Por otra parte, se encontró una excelente tendencia en las áreas evaluadas de desarrollo mediante la escala Abreviada de Desarrollo y las destrezas de ejecución en los infantes con zikagestacional de 3, 4 y 5 años, en la (Tabla 3) se muestra las ponderaciones de las escalas en cada área, así como sus promedios de medias, las desviaciones estándar, ítem mínimo y máximo de edad madurativa. Igualmente, se da la asimetría para conocer la distribución de los valores sean positivo o negativo con respectos a la media, así como la cantidad de datos que se concentran en la media (curtosis).

**Tabla 3:** Medida de tendencia central y dispersión para las áreas evaluadas de desarrollo mediante EAD y destrezas de ejecución para infantes con zika gestacional de 3, 4 y 5 años.

	Escala Abreviada de Desarrollo (EAD)				Destrezas de ejecución			
	Motricidad gruesa	Motricidad fina	Audición y lenguaje	Personal social	Destrezas motoras y praxis	Destrezas sensoriales perceptuales	Destrezas cognitivas	Destrezas regulación emocional	Destrezas comunicativas y sociales
Niños de 3 años con zika gestacional	4	0	0	0	0	0	0	0	0
	5	4	7	4	0	1	0	0	0
	6	4	7	5	0	1	0	0	0
	6	4	7	6	0	2	0	0	0
	7	4	7	7	0	2	0	0	0
	7	4	7	9	0	2	0	1	0
	9	6	9	10	0	2	0	1	0
	9	6	9	10	0	2	0	1	0
	9	9	10	10	0	2	0	1	0
	11	11	13	17	2	4	2	0	0
Sumatoria	73	52	76	78	2	18	2	4	0
N	10	10	10	10	10	10	10	10	10
Media Desv. Tip	7,30	5,20	7,60	7,80	0,20	1,80	0,20	0,40	0,00
	2,16	3,05	3,31	4,57	0,63	1,03	0,63	0,52	0,00
Mediana	7,00	4,00	7,00	8,00	0,00	2,00	0,00	0,00	0,00
Ítem Mínimo:	4,00	0,00	0,00	0,00	0,00	0,00	0,00	0,00	0,00
Ítem Máximo :	11,00	11,00	13,00	17,00	2,00	4,00	2,00	1,00	0,00
Asimetría de Pearson	0,42	1,18	0,54	-0,13	0,95	-0,58	0,95	2,32	-
Curtosis	0,24	0,13	0,09	0,17	0,00	0,14	0,00	5,00	-
Cuartil 1 2,75	5,75	4	7	4,75	0	1	0	0	0
Cuartil 3 8,25	9	6,75	9,25	10	0	2	0	1	0
Percentil 1,1 10	4,1	0,4	0,7	0,4	0	0,1	0	0	0
Percentil 9,9 90	10,8	10,8	12,7	16,3	1,8	3,8	1,8	0,1	0
Niños y niñas de 4 años con zika gestacional	Parte a- motricidad Gruesa	Parte b- motricidad fina	Parte c- audición y lenguaje	Parte d- personal social	Destrezas motoras y praxis	Destrezas sensoriales perceptuales	Destrezas cognitivas	Destrezas regulación emocional	Destrezas comunicativas y sociales
	10	11	13	17	0	1	0	0	0
	11	11	13	17	0	1	0	0	0
Sumatoria N Media Desv. Tip	21	22	26	34	0	2	0	0	0
	2	2	2	2	2	2	2	2	2
	10,50	11,00	13,00	17,00	0,00	0,20	0,00	0,00	0,00
	0,71	0,00	0,00	0,00	0,00	0,00	0,00	0,00	0,00
Niños y niñas de 5 años con zika gestacional	Parte a - motricidad gruesa	Parte b- motricidad fina	Parte c- audición y lenguaje	Parte d- personal social	Destrezas motoras y praxis	Destrezas sensoriales perceptuales	Destrezas cognitivas	Destrezas regulación emocional	Destrezas comunicativas y sociales
	8	6	9	9	0	1	0	0	0
	9	6	9	9	0	1	0	0	0
	9	9	9	9	0	1	0	0	0
Sumatoria N	26	21	27	27	0	3	0	0	0
	3	3	3	3	3	3	3	3	3
Media	8,67	7,00	9,00	9,00	0,00	0,30	0,00	0,00	0,00
Desv. Tip	0,58	1,73	0,00	0,00	0,00	0,00	0,00	0,00	0,00

Según los criterios evaluados para los infantes de 3 años con zika gestacional, se observa que los valores de motricidad gruesa, motricidad fina, audición y lenguaje, así como, destrezas motoras y praxis, destrezas cognitivas, destrezas regulación emocional, destrezas comunicativas y sociales se encuentran sesgadamente a la derecha y se concentran lejos de la media, excepto el área personal y social y, destrezas sensoriales perceptuales que a distribución es aproximadamente sesgada y sus valores se encuentran muy lejos de la media.

**Figura 1:**
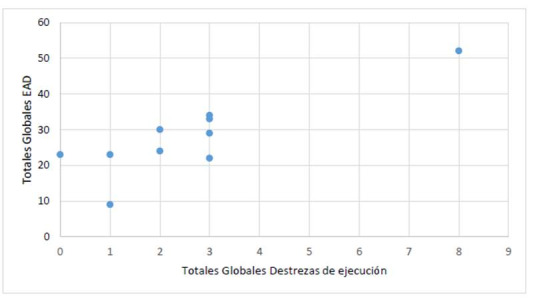
Diagrama de dispersión de Destrezas de ejecución y totales globales de EAD de infantes de 3 años con Zika Gestacional

Es decir, que los valores de curtosis y de asimetría para cada área evaluada con la EAD y destrezas de ejecución, permiten concluir que las puntuaciones tienen una distribución asimétrica a la derecha y leptocúrtica, excepto, la parte personal y social que es asimétrica a la izquierda y leptocúrtica. Mientras que destrezas sensoriales perceptuales indica que existe presencia de la minoría de datos en la parte izquierda de la media, aunque una tendencia de curtosis muestra una leve o débil concentración de los datos en torno a la media. Por otra parte, para los niños de 4 y 5 los datos no eran representativos, aunque siguen la misma tendencia que de los infantes de 3 años con zika gestacional y su correlación de las áreas evaluadas con respecto a la edad madurativa que se muestran en la (Tabla 4 y Figura 1)

**Tabla 4:** Correlación motricidad gruesa vs Destrezas motoras y praxis junto con Correlación de Totales Globales Destrezas de ejecución vs Totales Globales EAD la para infantes de 3 años con zika gestacional

Destrezas motoras y praxis		
Para n=10	R	P
Motricidad gruesa	0,601	0,066
Totales Globales EAD		
Para n=10	R	P
Totales Globales Destrezas de ejecución	0,853	0,002

En la (Tabla 4) la ponderación de la destreza motora y praxis encontró un coeficiente de correlación de 0,601 (moderada) y en la misma las destrezas de ejecución de los niños de 3 años con zika gestacional se obtuvo una correlación de 0,853 (fuerte)

## Discusión

La investigación permite ver en los resultados encontrados que los niños nacidos con la infección por virus zika durante la gestación tienen relación con otros diagnósticos y patologías que tienen un nivel de afectación en la edad madurativa y en las destrezas de ejecución en dichos infantes. De esta manera, los resultados concuerdan con el planteamiento de Arroyo^19^ quien señala que el espectro por virus Zika, incluye defectos congénitos asociados con microcefalia (pérdida auditiva y defectos oftalmológicos), así como epilepsia, discapacidad intelectual, entre otros; lo cual se demuestra en la (Tabla 2).

En ella se resalta que la edad madurativa de los infantes con zika gestacional, evidencian un rango de edad en la escala abreviada de desarrollo (EAD) muy diferente a la edad cronológica. Puesto que, dichos niños y niñas deberían tener, en una edad madurativa de 37 a 48 meses (53,3%). Mientras que un 20,0% está en una edad madurativa de 25 a 36 meses. El resto de los infantes, se encuentran entre 49 a 60 meses (un 13,3%) y los otros entre 61 a 72 meses (un 13,3%).

Sin embargo, en la misma Tabla 2, se demostró en la evaluación que la motricidad gruesa se encuentra entre el ítem mínimo de 4 meses y el ítem máximo de 11 meses, con una moda de mayor frecuencia en las distribuciones de datos de los infantes de 9 meses (rango de edad 7 a 9 meses según EAD), en el cual dichos niños apenas se sostienen sentado con ayuda; así como, se arrastra en posición prona y, se sientan por sí solo. En lo referente, a la motricidad fina, se conoció que los resultados hallados se encuentran entre cero (0) a 11 meses, con una moda de mayor frecuencia de datos de 4 meses, es decir, un rango de edad madurativa de 4 a 6 según EAD, en el cual, los infantes están en capacidad de agarrar objetos voluntariamente, sostener un objeto en cada mano y pasar objetos de una mano a otra.

En cuanto a la parte de audición y lenguaje, se logró aclarar que los hallazgos encontrados ubican un rango de cero (0) a 13 meses, con una moda de frecuencia de 7 meses. Esto indica que la mayoría de los infantes se encuentran con una edad madurativa de 7 a 9 meses según EAD, en el cual, los infantes están en la capacidad de pronunciar de 3 o más sílabas, hace sonar la campana y decir una palabra clara. Entretanto, lo concerniente a la evaluación de parte personal y social, se conoció que el ítem mínimo es de cero y el ítem máximo es de 17 meses, con una moda de frecuencia de 9 meses. Lo que indica que la mayoría de los infantes con zika gestacional se ubican en dicha área con una edad madurativa de 7 a 9 meses según EAD, donde ellos están en la capacidad de ayudar a sostener taza para beber, reaccionar a su imagen en el espejo e imitar aplausos.

De este modo, la Escala Abreviada de Desarrollo (EAD) es una herramienta para averiguar previamente anomalías globales y generales sobre las áreas motoras, de lenguaje, sociales y de coordinación. Debido que entre más lejano sea la edad madurativa de la cronológica, mayor es el grado de afectación que tiene el niño en su desarrollo. Además, los datos revelados en la Tabla 2, muestra que el desarrollo en edad cronológica no está siendo acorde con las etapas de la vida de los infantes infectados con zika gestacional, en la madurez de destrezas y habilidades.

Lo cual, coincide con el señalamiento del Ministerio de Salud de Argentina^20^ que diversas infecciones por *arbovirus* pueden afectar la edad madurativa de los niños y niñas y, con ello las destrezas ejecutivas, como se evidencia los hallazgos en la (Tabla 3) donde se dio a conocer las medidas de tendencia central y dispersión para las áreas evaluadas de desarrollo mediante EAD y destrezas de ejecución para infantes de 3, 4 y 5 años, y se encontró que en la motricidad gruesa para los infantes de 3 años, el valor promedio fue de 7,30 con una desviación estándar de 2,16 puntos, una mediana de 7 meses y el valor mínimo y máximo de 4 y 11 meses respectivamente.

Para la motricidad fina el valor medio hallado fue 5,20 y una desviación estándar de 3,05 puntos, con una mediana de 4 meses y un valor mínimo de 0 y máximo de 11. En cuanto al área audición y lenguaje, se halló que el valor medio fue de 7,60 con una desviación estándar de 3,31, con una mediana de 7 meses y el valor mínimo y máximo entre 0 y 13 meses respectivamente. En lo referente a al área personal y social, se encontró una media de 7,80 y una desviación estándar de 4,57 puntos. Con una mediana de 8 meses y un valor en meses mínimo de cero (0) y máximo de 17.

Como puede apreciarse, las áreas de menor progresión son la motora fina y motora gruesa, mientras que el área personal y social presenta un mejor ascenso evolutivo. Lo cual tiene una relación con las destrezas de ejecución, debido que las destrezas sensoriales perceptuales lograron un bajo valor medio con 1,8 y una desviación estándar de 1,03. Seguido de las destrezas motoras y praxis con una media de 2,0 y una desviación estándar de 0,63 punto que presentan una menor progresión en las destrezas ejecutivas para realizar acciones o comportamientos que utiliza el infante para localizar, identificar y responder sensaciones visuales y táctiles para mover e interaccionar físicamente con objetos.

Las otras destrezas ejecutivas adquirieron para destrezas cognitivas (media de 2,0 y desviación estándar de 0,63 puntos) y la destreza regulación emocional (media de 4,0 puntos y desviación estándar de 0,52 puntos). Las destrezas comunicativas y sociales obtuvieron un valor medio y desviación estándar de cero (0).

Esta últimas destrezas, concuerda con los hallazgos encontrados en la Tabla 2, donde se demostró que en audición y lenguaje el ítem mayor de maduración fue de 13 meses y una moda de frecuencia de 7 meses, por tal motivo, las destrezas comunicativas y sociales se encuentran un retraso en el desarrollo por los diversos afectaciones (microcefalia, retraso de desarrollo, parálisis cerebral, epilepsia, entre otros) que pareciera que son incapaces de cumplir las tareas típicas de su edad cronológica. Lo cual coincide con Jerez^24^ quien sostiene que la maduración y crecimiento va de la mano con la parte cognitiva, social, efectiva, lenguaje.

Todo lo anteriormente expuesto, infiere que los infantes con zika gestacional pudieran desarrollar muy pocas destrezas cognitivas, de comunicación de regulación de emociones, motoras (gruesa y fina) y sensoriales y perceptuales que permitan realizar las demanda de una actividad donde se contengan cosas y acciones mecánicas del cuerpo, así como solicitudes sociales para llevar a cabo la actividad, y las funciones y estructuras del cuerpo utilizado durante el desempeño de la actividad para infantes sanos cronológicamente de 3 años. Lo cual concuerda con la Sociedad Española de Ginecología y Sociedad Española de Infectología Pediátrica^17^ quienes sostienen que los infantes infectados por el virus zika producen trastornos en el desarrollo del sistema neurológico.

Lo anterior muestra que la motricidad gruesa de infantes de 3 años con zika gestacional correlaciona positivamente con las destrezas motoras y praxis de estos (ver tabla 4), donde el valor estadístico p (p=0,066< 0,05) se encuentra por encima de 0,05 y el coeficiente de correlación (r) para la motricidad gruesa y destrezas motoras y praxis es de 0,601 lo que muestra una correlación estadísticamente moderada entre estas dos áreas. De allí, que la correlación moderada entre los niños y niñas con infección por virus zika gestacional y las destrezas propias de la edad madurativa, desarrollen poco o nada la motricidad gruesa porque estas involucran resultados en las acciones y comportamiento de destrezas motoras y praxis.

De este modo, las destrezas de ejecución correlacionan positivamente con los resultados obtenidos de la EAD de los infantes de 3 años con zika gestacional, (*p< 0,05*). En ese sentido, el valor estadístico (*p=0,002*) se encuentra por debajo de 0,05 y el coeficiente de correlación (*r*) para estas dos variables es de 0,853 como se muestra en la (tabla 5) lo que indica una correlación estadísticamente fuerte entre ellas.

Por lo tanto, se demuestra que existe una correlación fuerte entre las variables infección por virus zika en niños y niñas, adquirida durante la gestación y las destrezas de ejecución propias de la edad madurativa. Lo cual indica que la destreza de ejecución involucra resultados la edad madurativa en las acciones y comportamientos de infantes con zika gestacional.

Por último, teniendo en cuenta la hipótesis de investigación plateada se acepta la hipótesis nula, ya que se ha encontrado que la infección por virus zika en niños y niñas, adquirida durante la gestación, limita fuertemente las destrezas de ejecución propias de la edad madurativa en esta población. Con lo cual se dio respuesta al tercer objetivo, ya que se determinó que existe una relación entre la infección por virus zika gestacional y las destrezas de ejecución, lo cual permiten describir y apreciar los ajustes desde el abordaje de la terapia ocupacional frente a las indagaciones y prácticas acerca de la capacidad de habilidades en el desempeño eficaz de los infantes con zika gestacional.

En el desarrollo de esta investigación se presentaron algunas limitantes asociadas con las diversas fases del proceso, por ejemplo, en la fase de identificación de los informantes, surgieron problemas para el acceso a las historias clínicas de las madres en las que se identificaba que durante su proceso de gestación habían sido afectadas por el Zika, que se complementó con el tamizaje que se les realizaba a los infantes al nacer para verificar la presencia del genotipo de esta patología.

Al momento de realizar el proceso de valoración a los infantes, fue necesario garantizar los recursos para desplazarlos a la universidad puesto que en el hospital dicha actividad no se pudo realizar. Finalmente, surge como otra limitante el poder consolidar un tamaño de muestra relativamente grande que cumpliera con los criterios de inclusión en la investigación. Se recomienda como futura investigación el desarrollo de procesos de rehabilitación tendientes a mejorar las condiciones de estos infantes afectados, como un complemento a este trabajo.

## Conclusiones

Con esta indagación se logra, no solo reconocer la EAD como instrumento útil para evaluar a los niños nacidos con Zika Gestacional, sino también la correlación que existe entre las destrezas y habilidades que manifiestan los infantes en las acciones o comportamientos que llevan a cabo en su ejecución y el desarrollo madurativo del mismo.

Las deducciones aquí encontradas aportan al seguimiento de la edad madurativa y desarrollo de los infantes con Zika Gestacional, a la disposición y sugerencia de terapeutas y los padres, al poder valorar si las destrezas motoras y praxis avanzan, así como las destrezas sensoriales preceptúales, de regulación emocional, la cognitiva, de comunicación y social en sus diferentes momentos evolutivos, de acuerdo con lo esperado para su edad cronológica en sus diferentes áreas motricidad gruesa fina, el lenguaje y el área personal-social y para su condición de desarrollo humano en todas sus dimensiones y proceso básicamente social y cultural.

De esta manera, existe una correlación para que los infantes con Zika Gestacional tengan una afectación fuerte en las acciones o comportamientos que la persona tenga para moverse e interactuar físicamente con actividades, objetos y por ende realizar una actividad motora aprendida. Pudiera ser que dichas acciones o comportamientos fueron afectados para localizar, identificar, seleccionar, asociar, organizar o recordar eventos sensoriales u otras destrezas que incluyen la parte cognitiva, de comunicación o social.

Precisamente este es el efecto de esta investigación a futuro; determinar la edad madurativa y las destrezas de ejecución de los niños y niñas priorizados del Programa Valientes del Futuro y población control mediante Escala Abreviada de Desarrollo y lista de chequeo, así como describir el nivel de afectación en dichas destrezas de ejecución y edad madurativa que están asociados a su proceso evolutivo de niños y niñas del programa valientes del futuro con la infección neonatal por virus Zika, especialmente en el área y destrezas antes mencionadas asociada al desarrollo evolutivo a futuro en estos niños en los hábitos, rutinas y comportamientos específicos y automáticos que pueden ser útiles o perjudiciales que puedan servir al profesional de terapia ocupacional con el fin de influir positivamente en la vida del cliente.
